# Hypoxia Conditioned Mesenchymal Stem Cell-Derived Extracellular Vesicles Induce Increased Vascular Tube Formation *in vitro*

**DOI:** 10.3389/fbioe.2019.00292

**Published:** 2019-10-23

**Authors:** Ciarra Almeria, René Weiss, Michelle Roy, Carla Tripisciano, Cornelia Kasper, Viktoria Weber, Dominik Egger

**Affiliations:** ^1^Department of Biotechnology, University of Natural Resources and Life Science, Vienna, Austria; ^2^Christian Doppler Laboratory for Innovative Therapy Approaches in Sepsis, Department for Biomedical Research, Danube University Krems, Krems, Austria

**Keywords:** mesenchymal stem cells, extracellular vesicles, hypoxia, angiogenesis, tube formation, therapeutic potential

## Abstract

Mesenchymal stem/stromal cells (MSCs) display a variety of therapeutically relevant effects, such as the induction of angiogenesis, particularly under hypoxic conditions. It is generally recognized that MSCs exert their effects by secretion of paracrine factors and by stimulation of host cells. Furthermore, there is increasing evidence that some therapeutically relevant effects of MSCs are mediated by MSC-derived extracellular vesicles (EVs). Since our current knowledge on MSC-derived EVs released under hypoxic conditions is very limited, we aimed to characterize MSC-derived EVs from normoxic vs. hypoxic conditions (5% O_2_). Adipose-derived MSCs were grown under normoxic and hypoxic conditions, and EVs were analyzed by flow cytometry using lactadherin as a marker for EVs exposing phosphatidylserine, CD63 and CD81 as EV markers, as well as CD73 and CD90 as MSC surface markers. Particle concentration and size distribution were measured by nanoparticle tracking analysis (NTA), and the EV surface antigen signature was characterized using bead-based multiplex flow cytometry. Furthermore, we evaluated the potential of MSC-derived EVs obtained under hypoxic conditions to support angiogenesis using an *in vitro* assay with an hTERT-immortalized human umbilical vein endothelial cell (HUVEC) line. Proliferation and viability of MSCs were increased under hypoxic conditions. EV concentration, size, and surface signature did not differ significantly between normoxic and hypoxic conditions, with the exception of CD44, which was significantly upregulated on normoxic EVs. EVs from hypoxic conditions exhibited increased tube formation as compared to normoxic EVs or to the corresponding supernatants from both groups, indicating that tube formation is facilitated by EVs rather than by soluble factors. In conclusion, hypoxia conditioned MSC-derived EVs appear to be functionally more potent than normoxic MSC-derived EVs regarding the induction of angiogenesis.

## Introduction

The application potential of mesenchymal stem cells (MSCs) in regenerative cell-based therapies has been gaining substantial interest (Squillaro et al., 2016; Mastrolia et al., 2019). Advances in MSC research have provided evidence that the therapeutic effects are largely independent of the physical proximity of administered MSCs to their target tissues, but can rather be attributed to trophic effects provided by MSCs upon secretion of EVs as well as soluble factors, such as cytokines and growth factors (Gnecchi et al., 2008; Karp and Teol, 2009; Wagner et al., 2009; Williams and Hare, 2011).

EVs are released by almost all cell types. Three EV subgroups have been discriminated according to their size, and biogenesis: (i) apoptotic bodies (>1,000 nm) released during early apoptosis; (ii) microvesicles (100 to 1,000 nm) formed via outward budding of the plasma membrane; and (iii) exosomes (40 to 100 nm) secreted after fusion of multivesicular bodies with the plasma membrane (Cocucci and Meldolesi, 2015). Since these subgroups can overlap in size, and as markers for their unambiguous discrimination are lacking, the generic term extracellular vesicles is used to describe both exosomes and MVs in the context of this study (Thery et al., 2018).

The heterogeneity of EVs mandates a combination of methods for their characterization (Yeo et al., 2013; Thery et al., 2018). Flow cytometry enables the detection and characterization of EVs with regard to the expression of EV markers (Thery et al., 2009; Wyss et al., 2014; Dragovic et al., 2015) and cellular origin (Weiss et al., 2018). Nanoparticle tracking analysis (NTA), commonly used to determine the average size distribution and number of particles in suspension based on their Brownian motion, has been adapted for the characterization of EVs. While NTA is well suited for screening purposes, it fails to provide information on EV-specific molecular properties (Dragovic et al., 2011; Sokolova et al., 2011). Genomic and proteomic profiling has demonstrated that EVs carry host-specific cargo, including mRNAs, miRNAs, lipids and proteins (Théry et al., 2002; Subra et al., 2007; Simpson et al., 2008), which can be transferred to recipient cells and alter their phenotype (Valadi et al., 2007). Multiplex bead-based flow cytometry has recently been introduced to characterize the surface marker profile of EVs, which mediates the interaction of EVs with their target cells (Wiklander et al., 2018) and is relevant to understand the molecular content and related functions of subsets of EVs and to identifying potential EV subsets with a defined therapeutic activity.

Previous reports have convincingly shown that the quality and therapeutic function of human MSCs are impacted by the isolation methods and culture conditions, as well as by the age and genetic traits of the donors (Dufrane, 2017; Liu et al., 2017). Several *in vitro* studies have demonstrated a significantly higher proliferative activity of MSCs cultured under hypoxia (1–10% O_2_) as compared to normoxia (21% O_2_) (Nekanti et al., 2010). Hypoxic preconditioning of MSCs generates distinctive changes in stem cell characteristics and influences the secretion of cytokines and growth factors (Kinnaird et al., 2004). It has therefore been suggested that the biological activity of MSC-derived EVs differs depending on the cell source and culture parameters, such as medium composition, oxygen content, duration of culture, as well as shear stress (Patel et al., 2017). Along this line, EV release from several human cancer cell lines was enhanced in hypoxia (1% O_2_) (Salomon et al., 2013; Endzelins et al., 2018; Kilic et al., 2018). Similar findings were reported for the release of EVs from hypoxic MSCs cultured in serum-free media (Lo Sicco et al., 2017), which was accompanied by an increased hypoxia-inducible factor 1-alpha (HIF-1-alpha) activation. It is therefore recognized that the cargo incorporated into EVs is regulated by hypoxic preconditioning, which ultimately affects their angiogenic potential, as well as their immunomodulatory and regenerative properties (King et al., 2012; Yu et al., 2012; Bian et al., 2014). Likewise, the EV surface protein profile may vary with the culture conditions, but studies on changes of EV surface proteins under normoxic/hypoxic conditions are lacking to date. Since EV surface molecules are crucial in mediating the interaction of EVs with their target cells, we characterized the surface signature of EVs from adipose-derived MSC culture supernatants generated under normoxic (21% O_2_) and hypoxic (5% O_2_) conditions and further investigated whether EVs derived from a hypoxic environment can increase vascular tube formation in HUVECs.

## Materials and Methods

### Cell Culture

The use of human tissue was approved by the ethics committee of the Medical University Vienna, Austria (EK Nr. 957/2011, 30 January 2013), and all donors gave written consent. Human MSCs were isolated within 8 h after surgery as previously described (Egger et al., 2017). MSCs from 6 donors (aged 20–70) were cultivated in standard medium composed of MEM alpha (Thermo Fisher Scientific, Waltham, MA, USA), 0.5% gentamycin (Lonza, Basel, Switzerland), 2.5% human platelet lysate (PL BioScience, Aachen, Germany; filtered through 0.2 μm filters according to the data sheet provided by the manufacturer; Supplementary Figure 1) and 1 IU/ml heparin (Ratiopharm, Ulm, Germany) in humidified atmosphere at 37°C, 5% CO_2_ and 21% or 5% O_2_, and cryo-preserved in liquid nitrogen as previously described (Neumann et al., 2014). Upon use, MSCs were thawed and subcultivated once, resulting in passage 2. Cells intended for cultivation at 5% O_2_ were isolated and subcultivated at 5% O_2_ until seeding. To characterize MSC-derived EVs, MSCs (passage 2) were seeded at a density of 3,000 cells/cm^2^ into 12-well plates (TPP, Trasadingen, Switzerland) (*n* = 4 each) and cultivated in 2 ml standard medium at 21 or 5% O_2_ for 6 days. The medium was completely exchanged every second day, and medium without cells served as control. The supernatants were stored at −20°C until further use.

### Viability Assay

MSCs were seeded into a 96-well plate at a density of 3,000 cells/cm^2^ and incubated at 21 or 5% O_2_ for 6 days. Viability was assessed using the resazurin-based TOX8 kit (Sigma Aldrich) according to the manufacturer's instructions. Fluorescence intensity at 560/590 nm was determined using a plate reader (Tecan, Männedorf, Switzerland) after 2 h incubation at 37°C (5 or 21% O_2_) with gentle shaking. The cell number was calculated based on a calibration curve.

### Isolation of Extracellular Vesicles

**Figure 1:**
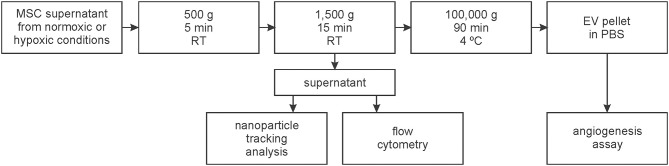
Workflow for the isolation and characterization of extracellular vesicles (EVs). EVs were isolated from the supernatant of mesenchymal stem cells (MSCs) which were cultivated at 21% O_2_ (normoxic) or at 5% O_2_ (hypoxic).

MSCs were cultured as described above. MSC supernatants were centrifuged at 500 g for 5 min and at 1,500 g for 15 min at room temperature (RT) in order to remove cells and debris. The resulting supernatant was stored at −20°C until further characterization by flow cytometry and NTA. EVs isolated from the supernatant of MSCs cultured for 72 h in vesicle-depleted standard medium under normoxic and hypoxic conditions were subjected to an additional centrifugation step at 100,000 g, 4°C for 90 min using a Sorvall WX 80 ultracentrifuge with SW 32 Ti rotor (Beckman Coulter Inc., CA). The resulting pellet was resuspended in 1 ml phosphate buffered saline (PBS; Thermo Fisher Scientific) and stored at −20°C prior to use in tube formation assays (Figure 1).

### Flow Cytometric Characterization of Stem Cell-Derived Extracellular Vesicles

Lactadherin was used as a marker for cell-surface derived EVs (Tripisciano et al., 2017; Weiss et al., 2018), since the large majority of the EV population that can be detected by flow cytometry (i.e., EVs larger than 250 nm) expose phosphatidylserine (PS). Cell culture supernatants were stained with FITC-conjugated lactadherin (LA) to detect PS, as well as with the following MSC surface markers: CD73-PE (Beckman Coulter, Brea, CA), CD90-APC-AF750 (Beckman Coulter), CD63-AF647 (Biolegend, San Diego, CA, USA) and CD81-PerCPCy 5.5 (Biolegend). CD41-PC7 (Beckman Coulter) was used as a marker for platelet-derived EVs. All antibodies used in this study and their respective fluorochromes are specified in Supplementary Table 1. Staining was performed for 15 min in the dark, and antibodies were centrifuged at 17,000 g for 10 min before use. Stained samples were diluted 5-fold in PBS, and analyzed on a Gallios flow cytometer (Beckman Coulter) equipped with 405, 488, and 638 nm lasers. Fluorescent-green silica particles (1.0, 0.5, 0.3 μm; excitation/emission 485/510 nm; Kisker Biotech, Steinfurt, Germany) were used for calibration, the triggering signal was set to forward scatter/size, and the EV gate was set below the 1 μm bead cloud as previously described (Tripisciano et al., 2017; Weiss et al., 2018) and as shown in **Figure 3A**. Data were acquired for 3 min at a flow rate of 10 μl/min and analyzed using the Kaluza Software (Beckman Coulter). EVs were identified as LA-positive events in the EV gate. To confirm that the signals in the EV fraction were indeed dependent on the presence of intact EVs, a detergent lysis control was included by treatment of the MSC supernatant with 0.25% TritonX-100 to lyse vesicles. Buffer controls, isotype controls, and single stainings of specific monoclonal antibodies are shown in Supplementary Figure 2.

### Nanoparticle Tracking Analysis

The particle concentration and size distribution of MSC-derived EVs was determined in cell culture supernatants using nanoparticle tracking analysis (NTA; Zeta View, Particle Metrix, Inning, Germany). Measurements were performed at RT using the following instrument settings: 80 (sensitivity), 1,000 (maximal area), 5 (minimal area), and 25 (brightness). Data were acquired in one cycle of measurement over 11 positions and were analyzed using the software ZetaView version 8.04.02.

### Bead-Based Multiplex Exosome Flow Cytometry Assay

MSCs were seeded at 3,000 cells/cm^2^ in passage 2 and cultivated for 72 h. The cell culture supernatants were subjected to bead-based multiplex EV analysis by flow cytometry (MACSPlex Exosome Kit, human; Miltenyi Biotec, Bergisch Gladbach, Germany). To obtain samples for EV characterization, supernatants were pre-cleared according to manufacturer's recommendation. Pre-cleared cell culture supernatants were incubated with 15 μl of MACSPlex Exosome Capture Beads containing 39 different antibody-coated bead subsets and with 15 μl MACSPlex Exosome Detection Reagent containing APC-conjugated CD9, CD63, and CD81 for 1 h with gentle agitation at RT. Beads were washed with 1 ml of MACSPlex Buffer prior to flow cytometric analysis (CytoFLEX LX, Beckman Coulter). Raw APC median fluorescence intensity (MFI) for all surface epitope capture bead subsets was corrected by subtracting the corresponding MFI values obtained for isotype control beads. All antibodies and the respective fluorochromes are specified in Supplementary Table 1.

### Angiogenesis Assay

MSCs from one donor (female, 28) were cultured in standard medium for 72 h under normoxic or hypoxic conditions at 37°C and 5% CO_2_ (*n* = 5). As EV fractions were normalized regarding their protein content (see below), the medium was centrifuged at 100,000 g for 14 h at 4°C prior to use in order to deplete any non-MSC-derived protein components derived from medium supplements. Culture supernatants were collected and subjected to serial centrifugation at 500 g for 5 min, for 1,500 g for 15 min, and for 90 min at 100,000 g at 4°C (Sorvall WX 80 ultracentrifuge, SW 32 Ti rotor, Beckman Coulter) to pellet EVs. The EV pellet was resuspended in PBS. For the tube formation assay, immortalized HUVECs (Schiller et al., 2009) were seeded at a density of 3.5 × 10^5^ cells/ml onto growth factor reduced Matrigel (Corning, New York, USA) in an ibidi μ-plate Angiogenesis 96 Well (Ibidi, Gräfelfing, Germany). HUVECs were treated with EVs (corresponding to 100 μg/ml protein), with EV-depleted supernatant (100 μg/ml protein; both from normoxic or hypoxic culture), with 100 ng/ml vascular endothelial growth factor (VEGF) in endothelial cell growth medium (EGM-2; Lonza) as positive control, and with PBS as negative control. Cells were incubated for 16 h and stained with calcein acetoxymethyl ester (calcein-AM, Sigma Aldrich). Fluorescence images were obtained at excitation/emission wavelengths of 490/515 nm with an inverted fluorescence microscope (DM IL LED by Leica, Wetzlar, Germany). The tube characteristics were analyzed and quantified using the Angiogenesis Analyzer toolset in ImageJ (version 1.52, NIH, Baltimore, MD, USA) (Carpentier, 2012).

### Statistical Analysis

Statistical analysis was performed using GraphPad Prism version 7.02 (La Jolla, CA, USA). Data are presented as mean ± standard deviation (SD). For multiple comparisons, repeated measures two-way ANOVA followed by Tukey's multiple comparisons test was used to assess the increase/decrease within one group over time (time effect), while Bonferroni's multiple comparisons test was used to assess the difference between normoxic/hypoxic conditions at a given time point (group effect). Significance was accepted at *p* ≤ 0.05.

## Results

### Cultivation of MSCs Under Normoxic and Hypoxic Conditions

**Figure 2:**
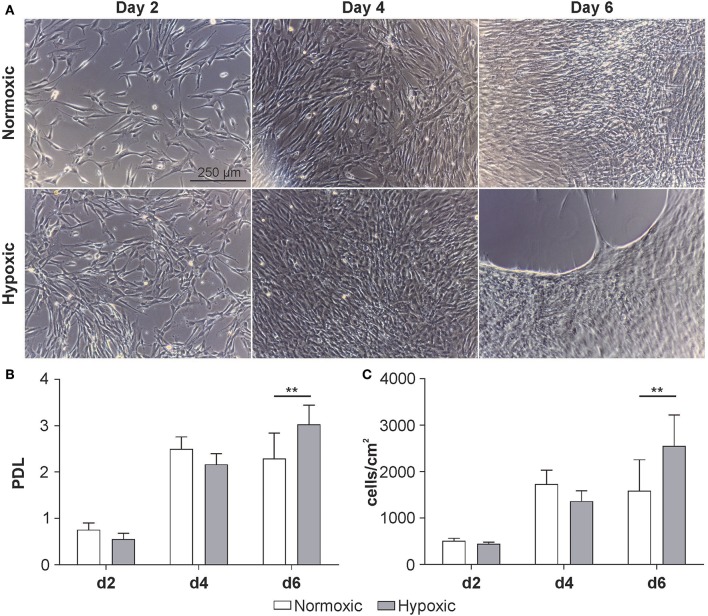
Growth kinetics of MSCs at passage 3 cultivated at 21 % O_2_ (normoxic) or 5 % O_2_ (hypoxic). **(A)** Representative micrographs, **(B)** population doubling level (PDL) and **(C)** cellular concentration at day 2, 4 and 6. Cell number was determined using a resazurin-based viability assay as described in Materials and Methods. Data are given as mean ± SD (*n* = 6) **p < 0.01.

MSCs were grown under normoxic or hypoxic conditions for 6 days. Micrographs of the cells suggest a higher proliferation rate of MSCs under hypoxic conditions. After reaching confluence on day 4, the cells started to detach by day 6 (Figure 2A). Both, the population doubling level (PDL) (normoxic 2.3 ± 0.5 vs. hypoxic 3.0 ± 0.4; Figure 2B) and the cell density were significantly higher under hypoxic conditions (normoxic 1.6 × 10^5^ ± 3.4 × 10^4^ vs. hypoxic 2.2 × 10^5^ ± 7.3 × 10^4^ cells/cm^2^; Figure 2C).

### Flow Cytometric Characterization of MSC-Derived EVs Under Normoxic and Hypoxic Conditions

**Figure 3:**
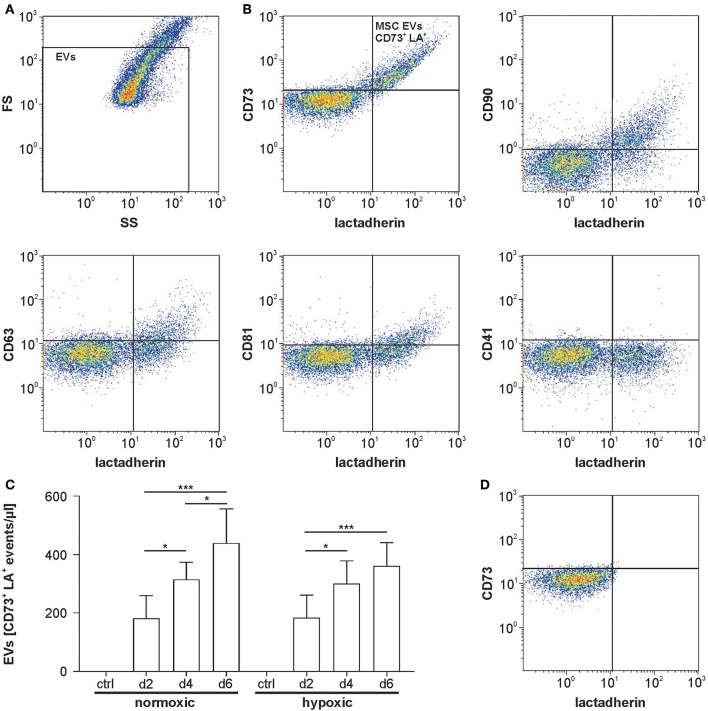
Flow cytometric characterization of MSC-derived EVs. **(A)** Flow cytometric characterization was performed with fluorescent-green silica particles, and the EV gate was set below the 1 μm bead cloud. A forward scatter vs. side scatter (FS vs. SS) dot plot for MSC supernatant is shown as example. **(B)** MSC supernatants were stained with FITC-conjugated lactadherin as marker of phosphatidylserine-exposing EVs as well as with the general EV markers CD63 and CD81 and with the MSC markers CD73 and CD90. CD41 was used as a negative marker to label platelet-derived EVs potentially derived from human platelet lysate used as medium supplement. Lactadherin vs. surface marker plots are shown. **(C)** Release of MSC-derived EVs under normoxic or hypoxic conditions over time (*n* = 6). **(D)** MSC supernatant was treated with 0.25% TritonX-100 during staining as detergent lysis control, abolishing all signals in the EV gate and confirming the presence of vesicles. Data are given as mean ± SD. *p < 0.05; ***p < 0.001.

According to flow cytometry, 100 and 75% of all EVs were positive for the MSC markers CD73 and CD90, respectively, while 35 and 40% were positive for the tetraspanins CD63 or CD81. Staining with CD41, which was included as control, did not provide evidence for the presence of EVs derived from the platelet lysate used as medium supplement (Figure 3B). Detergent lysis by treatment of MSC culture supernatants with 0.25% Triton X-100 abolished all signals in the EV gate, confirming the presence of intact vesicles (Figure 3D). Increased release of EVs was observed over time with a maximum after 6 days of cultivation. Supernatants from MSCs grown under normoxic or hypoxic conditions did not differ regarding the EV concentration (Figure 3C).

### Characterization of MSC-Derived EVs by Nanoparticle Tracking Analysis

**Figure 4:**
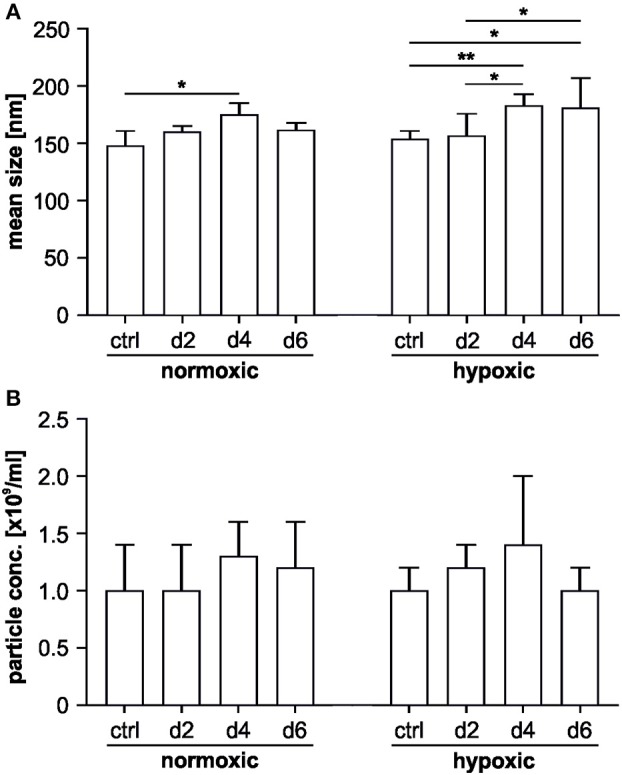
Nanoparticle tracking analysis of MSC-derived EVs. The characterization was performed in scatter mode as described in Materials and Methods. **(A)** Mean particle sizes of MSC-derived EVs under normoxic or hypoxic conditions on day 2, 4 and 6; **(B)** mean number of particles/ml in MSC supernatants under normoxic or hypoxic conditions on day 2, 4, and 6. Data are presented as mean ± SD (*n* = 6; *p < 0.05; **p < 0.01).

According to NTA, supernatants from MSCs grown under normoxic and hypoxic conditions did not differ with regard to mean particle size and concentration. Mean particle size increased over time for both groups, while we failed to detect an increase in total particle concentration over time for the individual groups (Figure 4).

### Detection of EV Surface Signatures

**Figure 5:**
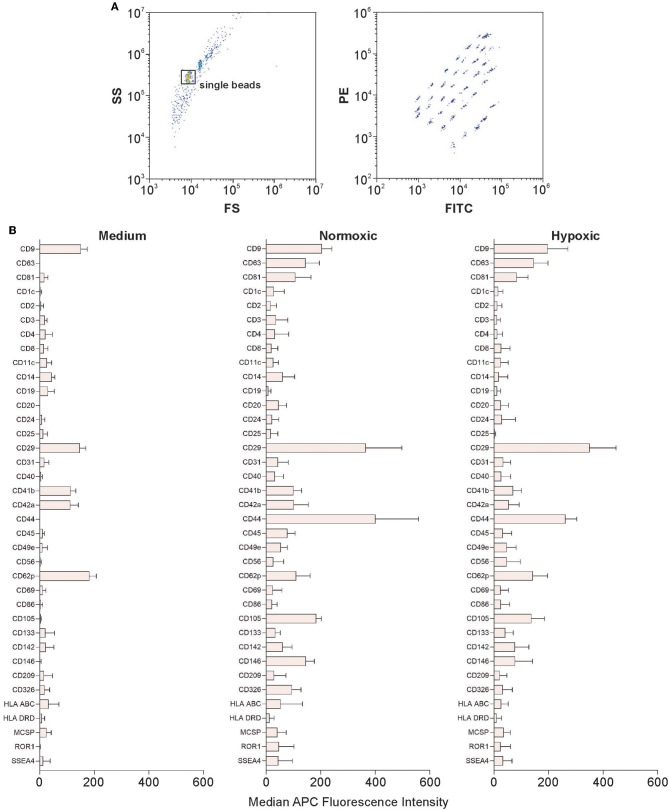
Characterization of EVs using multiplex bead-based flow cytometry. Analysis was performed using a CytoFLEX LX flow cytometer as described in Materials and Methods. **(A)** Gating strategy to identify single beads and discrimination of differently labeled bead populations identified by their fluorescence in the FITC vs. PE channel. **(B)** Identification of EV surface signatures in MSC culture supernatants from normoxic or hypoxic conditions; medium without cells served as a control. A representative quantification of the median APC fluorescence values for all 37 surface epitope bead populations after isotype correction is shown. The difference in expression between normoxic and hypoxic conditions was statistically significant only for CD44. Data are given as mean ± SD (*n* = 4).

The bead-based multiplex assay used to characterize EV surface signatures comprises 37 labeled capture bead populations, each of them coated with different monoclonal antibodies against individual EV surface antigens. Individual bead populations can be identified and gated based on their respective fluorescence intensity (Figure 5A). After incubation with MSC culture supernatants, bead-captured EVs are detected by counterstaining with APC-labeled antibodies targeting the EV tetraspanins CD9, CD63 and CD81.

Platelet markers including CD49e (integrin α-5), CD9 (tetraspanin 29), CD62p (P-selectin), CD42a (glycoprotein IX) and CD29 (integrin β-1) were detected in the control medium containing 2.5% human platelet lysate. The MSC markers CD105 (endoglin, a receptor for transforming growth factor beta, TGF-β), CD63 and CD81 (tetraspanins), as well as CD146 (melanoma cell adhesion molecule) were detected at intermediate-positive APC fluorescence intensity for both, normoxic and hypoxic EVs. CD29 was present at high-positive APC fluorescence intensity on EVs from both, normoxic and hypoxic conditions. CD44, a surface glycoprotein involved in cell adhesion and migration, was expressed at significantly higher levels under normoxic as compared to hypoxic conditions (Figure 5B).

### Characterization of Angiogenic Properties of MSC-Derived EVs From Normoxic and Hypoxic Conditions

**Figure 6:**
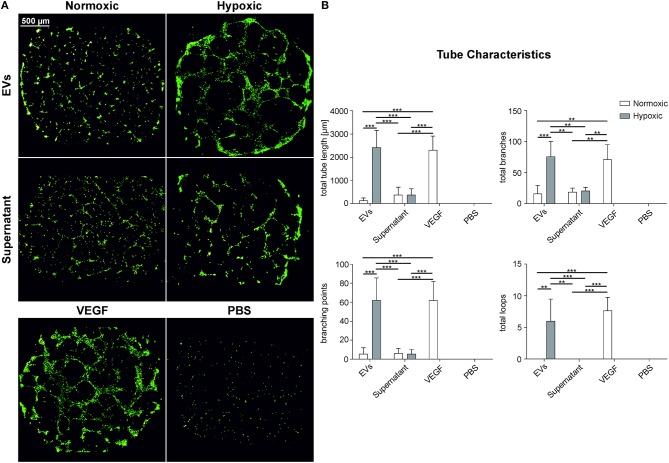
Angiogenic properties of MSC-derived EVs obtained under normoxic and hypoxic conditions. HUVECs on growth factor reduced Matrigel were incubated with EVs derived from MSCs grown under normoxic and hypoxic conditions (100 μg protein/ml) as well as with EV-depleted supernatant. **(A)** HUVECs stained with calcein-AM and **(B)** tube characteristics of HUVECs after 16 h of incubation with the EV preparation, the corresponding supernatant, 100 ng/ml VEGF in EGM-2 (VEGF, positive control) and PBS (negative control). Data are given as mean ± SD (*n* = 3; **p < 0.01; ***p < 0.001).

We used a HUVEC tube formation assay to investigate the capacity of MSC-derived EVs to induce vascular tube formation. We employed an immortalized HUVEC cell line to increase the reproducibility, and seeded HUVECs onto growth factor-reduced Matrigel to avoid induction of tube formation by growth factors. The total tube length (length of all branches and loops), the number of branches, branching points, and loops was significantly increased (*p* < 0.01) after incubation with EVs from hypoxic conditions, as compared to EVs from normoxic conditions, and as compared to the EV-depleted supernatants from normoxic and hypoxic conditions (Figure 6). A rearrangement of HUVECs was observed for the EV-depleted supernatants, but without formation of complete tubes. Still, the tube formation parameters were higher than in the negative control.

## Discussion

A number of beneficial effects has been observed upon cultivation of MSCs under hypoxic conditions, usually at 1–10 % O_2_ (Lavrentieva et al., 2010). Furthermore, the therapeutic potential of MSC-derived EVs has been shown in the field of kidney, heart, lung, as well as brain diseases (Börger et al., 2017; Phinney and Pittenger, 2017).

Our current understanding is that MSC-derived EVs can mediate their effects mainly by horizontal transfer of mRNAs, miRNAs, and proteins to their target cells, and that this cargo is affected by cell culture conditions (Phan et al., 2018). Next to the EV cargo, EV surface signatures are crucial for mediating the function of EVs, since they determine the interaction of EVs with their specific target cells.

In this study, we therefore aimed to characterize EVs derived from MSCs under normoxic and hypoxic conditions. We focused on potential differences regarding EV surface signatures as well as on differences related to angiogenesis, since enhanced angiogenesis of human umbilical vein endothelial cells has been previously described upon stimulation with exosome-like vesicles (Dai et al., 2017).

We used a combination of markers for the flow cytometric characterization of MSC-derived EVs. Lactadherin served as a marker for EVs, since the large majority of EVs detectable in flow cytometry are known to be derived from the cell surface and to expose phospatidylserine. The tetraspanins CD63 and CD81 were used as additional EV markers, complemented by the MSC markers CD73 and CD90. CD41 was added as a marker for EVs potentially derived from the human platelet lysate that served as medium supplement. We found that, while the large majority of PS-exposing EVs were CD73^+^ or CD90^+^, <50% of all PS-exposing EVs were CD63^+^ or CD81^+^, most likely because the tetraspanins CD63 and CD81 are mainly associated with exosomes. Since the current detection limit for EVs in flow cytometry is about 250 nm (Saunderson et al., 2008; Crescitelli et al., 2013; Willms et al., 2018), smaller CD63^+^ or CD81^+^ EVs, including the majority of exosomes, remain undetected. Flow cytometry did not provide evidence for platelet-derived CD41^+^ EVs in a size range above 250 nm, consistent with the fact that human platelet lysate was filtered (0.2 μm) prior to use.

With the flow cytometry protocol used in this study, the concentration of MSC-derived EVs (LA^+^CD73^+^CD90^+^CD63^+^CD41^−^) was in the range of 2–4 × 10^5^ EVs/ml. Other studies have reported EV concentrations ranging from 1 × 10^8^ to 1 × 10^11^ EVs/ml, depending on the EV source, as well as the isolation and flow cytometry protocols used (Tripisciano et al., 2017; Endzelins et al., 2018; Reis et al., 2018; Weiss et al., 2018). The release of EVs in our study increased over time, correlating with the increasing cell number, in agreement with previous reports that frequent collection of cell culture supernatant enhances the yield of EVs (Patel et al., 2017). Moreover, there is evidence that both, EV release and cell growth are reduced at higher initial seeding densities, supporting the notion that EVs are released to support intercellular communication in the culture environment (Patel et al., 2017; Ohyashiki et al., 2018).

Complementing flow cytometry, we used NTA to determine particle concentrations in MSC culture supernatants. Analysis in scatter mode yielded particle concentrations in the range of 1 × 10^9^ EVs/ml, more than 3 orders of magnitude higher as compared to flow cytometry. While this difference can be partly attributed to enhanced detection of smaller EVs using NTA, it also reflects the presence of non-vesicular particulate material derived from the cell culture medium (Lane et al., 2017), since NTA in scatter mode detects any light scattering event and thus is considerably less specific than flow cytometry. NTA also revealed a significant increase in the particle mean size over time. The particle size was comparable to previously published studies with a mean size ranging from 50 to 200 nm (Reis et al., 2018; Valandani et al., 2018; Li et al., 2019; Witwer et al., 2019).

Bead-based multiplex flow cytometry used to characterize EV surface signatures (Wiklander et al., 2018) did not indicate differences for vesicular (CD63, CD81) or MSC markers under normoxic vs. hypoxic conditions. As an exception, CD44, an MSC glycoprotein involved in cell-cell interaction, cell adhesion, and migration (Ramos et al., 2016), was significantly upregulated on EVs obtained under normoxic as compared to hypoxic conditions. CD44 expression on MSC-derived EVs has been previously shown to be required for their uptake by target cells (Bruno et al., 2009; Monsel et al., 2015). Moreover, CD44 expressing EVs have been recently implicated in anti-inflammatory effects, since the prevention of CD44^+^ EV uptake by monocyte-derived macrophages (MDM) using an anti-CD44 antibody abrogated the ability of MSC-conditioned medium (and thus of MSC-derived EVs) to reduce macrophage TNF secretion (Morrison et al., 2017). However, based on our current data, we are not able to link the enhanced CD44 expression on normoxic EVs to the observed differences in angiogenesis induced by normoxic vs. hypoxic EVs.

Bead-based multiplex flow cytometry also revealed the presence of platelet-derived markers, specifically CD62p, in MSC culture supernatants, likely due to the use of human platelet lysate as cell culture supplement. While filtered (0.2 μm) platelet lysate was used, which did not contain detectable amounts of EVs in a size range above 250 nm according to flow cytometry, smaller platelet-derived EVs (or even soluble CD62p) remained undetected in flow cytometry, but were caught by beads coated with anti-CD62p and therefore detected in bead-based flow cytometry.

To investigate potential functional differences for normoxic and hypoxic MSC-derived EVs, we focused on their ability to support angiogenesis. MSCs and MSC-conditioned cell culture supernatants are known to induce vascular tube formation of HUVECs (Sorrell et al., 2009). Apparently, this paracrine effect is not only caused by soluble growth factors, such as fibroblast growth factor (FGF) and VEGF, or by cytokines, but also by specific factors associated with EVs (Nakamura et al., 2015; Merino-Gonzalez et al., 2016). MSC-derived EVs are known to stimulate angiogenesis (Lopatina et al., 2014) despite their low levels of VEGF (Nakamura et al., 2015). Furthermore, it is known that MSCs and MSC culture supernatants from hypoxic conditions promote angiogenic effects, which are commonly assessed by the tube formation of HUVECs (Stubbs et al., 2012; Hsiao et al., 2013). Still, it has not been previously addressed whether similar effects are induced by MSC-derived EVs obtained under hypoxic conditions. Incubation of HUVECs with purified MSC-derived EVs or the corresponding EV-depleted MSC culture supernatants significantly increased vascular tube formation for EVs from hypoxic conditions. The induction was comparable to the positive control, 100 ng/ml VEGF in EGM-2 containing a mixture of growth factors including FGF, epidermal growth factor (EGF) and insulin-like growth factor (IGF). MSC-derived EVs from normoxic conditions, in contrast, induced the formation of only a small number of branches, but no intact loops, which is not consistent with a previous study in which normoxic EVs significantly increased tube formation as compared to the EV-depleted MSC culture supernatant (Lopatina et al., 2014; Nakamura et al., 2015).

Overall, hypoxic conditioning of MSCs appears to increase vascular tube formation induced by MSC-derived EVs, suggesting that the induction of vascular tube formation by MSC-derived EVs is mainly mediated by EVs and to a lesser extent by soluble growth factors or cytokines present in the culture supernatant. This is in line with recent findings that angiogenic effects are mainly promoted by the transfer of miRNAs, such as miR-21, miR-23a, miR-125a, miR-126, and miR-130a (Liang et al., 2016; Gong et al., 2017; Zimta et al., 2019).

While our data indicate that hypoxic preconditioning of MSCs can promote the ability of the corresponding MSC-derived EVs to induce vascular tube formation, further effects of hypoxic preconditioning remain to be elucidated. Hypoxic preconditioning might also increase other effects that are usually enhanced in MSCs from hypoxic conditions, such as chondrogenesis (Malladi et al., 2006), immunosuppressive properties (Roemeling-van Rhijn et al., 2013), or the expression of regenerative growth factors (Wei et al., 2012; Chang et al., 2013).

Our findings highlight the relevance of culture conditions for the generation and composition of MSC-derived EVs, which has already been subject of a number of studies (Phan et al., 2018). MSCs were shown to produce more EVs when cultivated as 3D aggregates as compared to 2D cultivation, and these EVs displayed enhanced angiogenic and neurogenic potential. These effects were further promoted by cultivation of 3D aggregates under dynamic conditions (Cha et al., 2018), and the generation of EVs was elevated when MSCs were grown on a 3D collagen matrix (Tao et al., 2017). Apparently, physiological culture conditions can increase both, the therapeutic potential of MSCs and the potency of MSC-derived EVs. In this context, bioreactor systems for the expansion of MSCs in 3D aggregates under hypoxic conditions (Egger et al., 2017) are suited to generate and control physiological culture conditions. While the efficiency and the potency of EVs produced in these systems need to be subject of future studies, bioreactor systems will probably play key roles in the stable, large-scale production of MSC-EVs under physiologic conditions (Egger et al., 2018).

## Conclusions

In this study, we characterized MSC-derived EVs from hypoxic conditions in comparison to normoxic MSC-EVs. We found that hypoxia conditioned MSC-EVs induced significantly increased epithelial tube formation when compared to normoxic EVs. This effect was largely mediated by EVs and not by other soluble factors, suggesting that hypoxic conditioning might be used to increase the therapeutic potential of MSC-EVs.

## Data Availability Statement

The raw data supporting the conclusions of this manuscript will be made available by the authors, without undue reservation, to any qualified researcher.

## Ethics Statement

The studies involving human participants were reviewed and approved by Ethics committee of the Medical University Vienna, Austria. The patients/participants provided their written informed consent to participate in this study.

## Author Contributions

CA performed cell culture experiments and tube formation assays and wrote the manuscript. RW designed and performed flow cytometry experiments, interpreted the data, and wrote the manuscript. MR performed cell culture experiments. CT performed NTA measurements and contributed to interpretation of the data and to writing the manuscript. CK and VW designed the study and reviewed the manuscript. DE designed the cell culture experiments, interpreted the data, and wrote the manuscript. All authors read and approved the final manuscript.

### Conflict of Interest

The authors declare that the research was conducted in the absence of any commercial or financial relationships that could be construed as a potential conflict of interest.

## Abbreviations

EVs: Extracellular vesicles
HUVECs: human umbilical vein endothelial cells
LA: lactadherin
MFI: median fluorescence intensity
MSCs: mesenchymal stem cells
NTA: nanoparticle tracking analysis
PS: phosphatidylserine
VEGF: vascular endothelial growth factor.
