# Supplementary material for: Hypoxia Conditioned Mesenchymal Stem Cell-Derived Extracellular Vesicles Induce Increased Vascular Tube Formation in vitro
Source: Front Bioeng Biotechnol. 2019 Oct 23;7:292. doi: 10.3389/fbioe.2019.00292 (PMC6819375; doi:10.3389/fbioe.2019.00292)
Supplement: Supplementary file 1 [file Table_1.DOCX]

**Supplementary Table 1 S1:** Fluorochrome-labeled antibodies used in this study.

| **Flow Cytometry** | | | | | | |
| --- | --- | --- | --- | --- | --- | --- |
| Antigen | Clone | Marker for | Fluorochrome | | Abbreviation | Supplier |
| CD73 | AD-2 | MSC | Phycoerythrin | | PE | Beckman Coulter |
| CD90 | Thy-1/310 | MSC | Allophycocyanin Alexa Fluor 750 | | APC AF750 | Beckman Coulter |
| CD63 | H5C6 | tetraspanin | Alexa Fluor 647 | | AF647 | Biolegend |
| CD81 | 5A6 | tetraspanin | Peridinin-Chlorophyll Protein Cyanin 5.5 | | PerCP/Cy5.5 | Biolegend |
| CD41 | P2 | platelets | Phycoerythrin Cyanin 7 | | PC7 | Beckman Coulter |
| lactadherin | - | phosphatidylserine | Fluorescein Isothiocyanate | | FITC | Haematologic Technologies, Inc. |
| **Bead-Based Multiplex Exosome Flow Cytometry: Capture Antibodies** | | | | | | |
| Antigen | Clone | Marker for | Antigen | Clone | | Marker for |
| CD9 | SN4 | tetraspanin, platelets | CD44 | DB105.2G12.1.6 | | MSC |
| CD63 | H5C6 | tetraspanin | CD45 | 5B1 | | leukocytes |
| CD81 | REA513 | tetraspanin | CD49e | NKI-SAM-1 | | thymocytes, lymphocytes |
| CD1c | AD5-8E7.9.4 | antigen presenting cells | CD56 | REA196 | | NK cells |
| CD2 | LT2.2 | T cells, NK cells | CD62p | REA389 | | platelets |
| CD3 | BW264/56 | T cells | CD69 | FN50 | | hematopoietic stem cells, T cells |
| CD4 | VIT4.3 | T cells, monocytes | CD86 | FM95 | | antigen presenting cells |
| CD8 | BW135/80BMA081 | T cells | CD105 | 43A4E1.71 | | endoglin, MSC |
| CD11c | MJ4-27G12.4.6 | dendritic cells | CD133 | AC133.1.6.2.1 | | cancer stem cells |
| CD14 | TÜK4 | monocytes | CD142 | HTF1-7B8 | | tissue factor |
| CD19 | LT19 | B cells | CD146 | 541/10B2 | | MSC |
| CD20 | LT20.B4 | B cells | CD209 | DCN-47.5.4 | | macrophages, dendritic cells |
| CD24 | 32D12 | B cells, neutrophils | CD326 | HEA125 | | epithelial cells |
| CD25 | 3G10 | T cells | HLA ABC | REA230 | | human leukocyte antigen |
| CD29 | TS2/16.2.1 | MSC, platelets | HLA DRD | REA332 | | human leukocyte antigen |
| CD31 | AC128 | endothelial cells | MCSP | EP1 | | melanoma cells |
| CD40 | HB14 | antigen presenting cells | ROR1 | 2A2 | | receptor tyrosine kinase |
| CD41b | REA336 | platelets | SSEA4 | REA101 | | embryonic stem cells |
| CD42a | REA209 | platelets |  |  | |  |
| **Bead-Based Multiplex Exosome Flow Cytometry: Detection Antibodies** | | | | | | |
| Antigen | Clone | Marker for | Fluorochrome | Abbreviation | | Supplier |
| CD9 | SN4 | tetraspanin | Allophycocyanin | APC | | Miltenyi |
| CD63 | H5C6 | tetraspanin | Allophycocyanin | APC | | Miltenyi |
| CD81 | REA513 | tetraspanin | Allophycocyanin | APC | | Miltenyi |
