# Supplementary material for: Hypoxia Conditioned Mesenchymal Stem Cell-Derived Extracellular Vesicles Induce Increased Vascular Tube Formation in vitro
Source: Front Bioeng Biotechnol. 2019 Oct 23;7:292. doi: 10.3389/fbioe.2019.00292 (PMC6819375; doi:10.3389/fbioe.2019.00292)
Supplement: Supplementary file 2 [file Data_Sheet_1.PDF]

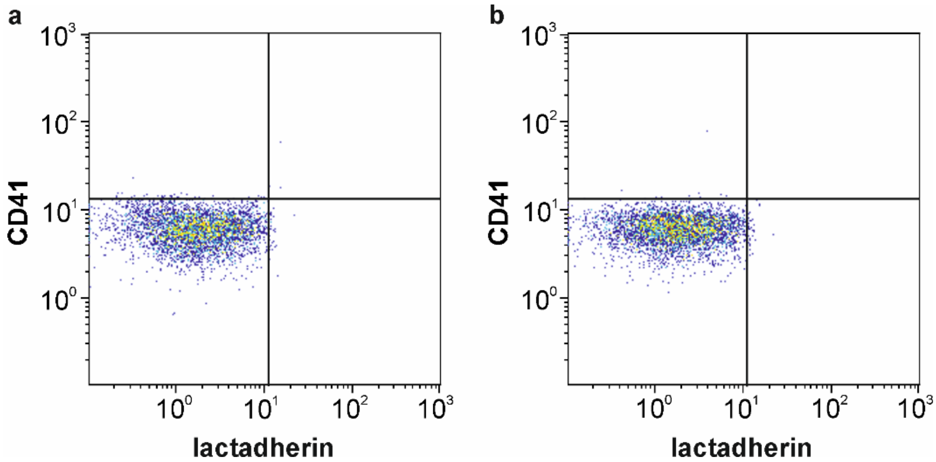

Supplementary Figure S1. Flow cytometric analysis of the human platelet lysate used in this study, (a) without and (b) with additional filtration using an 0.2 µm filter. CD41 was used as marker for platelet origin and lactadherin was employed to detect phosphatidylserine-exposing extracellular vesicles as described in the main manuscript. The absence of EVs in both samples confirms that the product was pre-filtered by the supplier.
