# Supplementary material for: Hypoxia Conditioned Mesenchymal Stem Cell-Derived Extracellular Vesicles Induce Increased Vascular Tube Formation in vitro
Source: Front Bioeng Biotechnol. 2019 Oct 23;7:292. doi: 10.3389/fbioe.2019.00292 (PMC6819375; doi:10.3389/fbioe.2019.00292)
Supplement: Supplementary file 3 [file Data_Sheet_2.PDF]

Supplementary Material

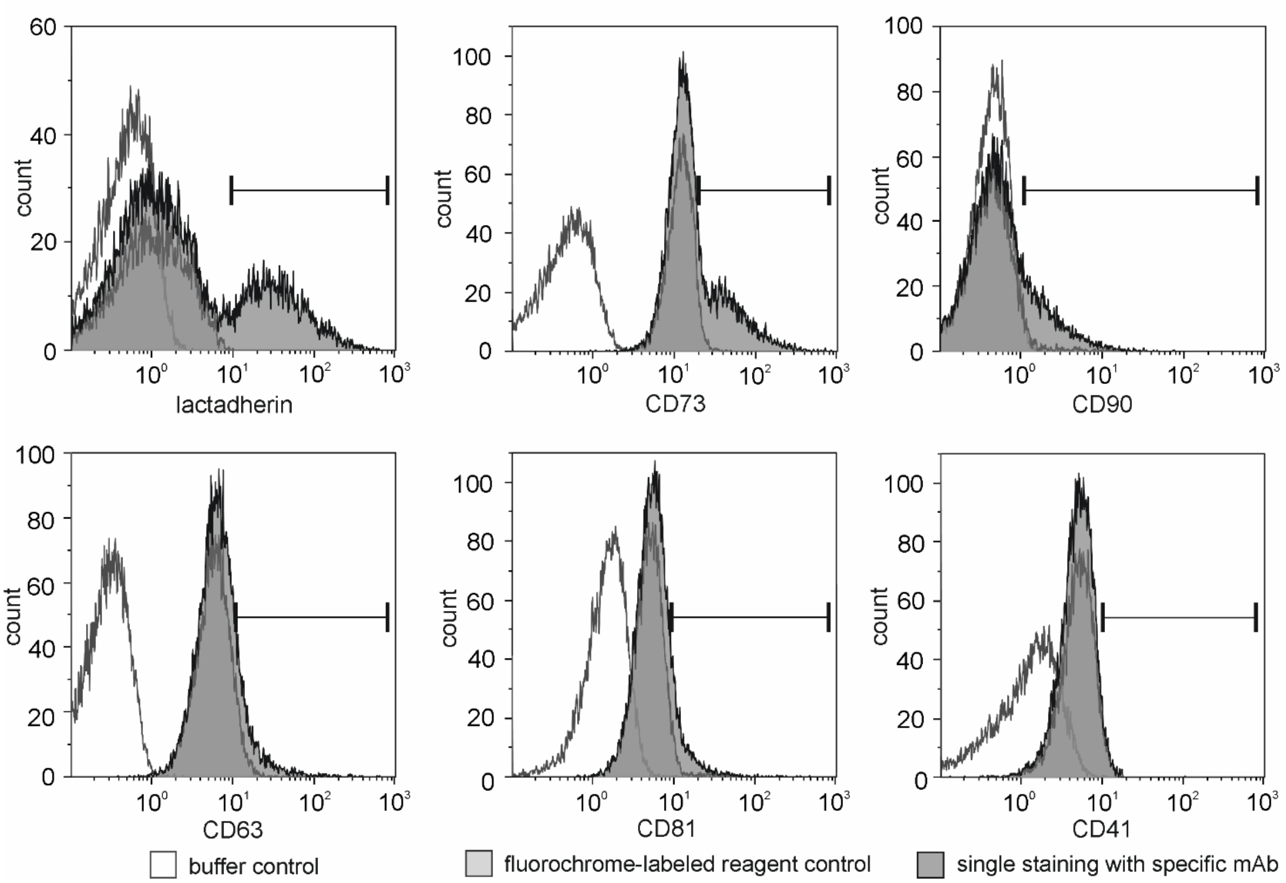

Supplementary Figure S2: Controls for the flow cytometric characterization of MSC-derived EVs. Cell culture supernatants were stained with lactadherin as marker for phosphatidylserine, with the general EV markers CD63 and CD81, as well as with the MSC markers CD73 and CD90 as described in Materials and Methods. CD41 was used as negative marker to label platelet-derived EVs derived from human platelet lysate contained in the culture medium. The respective buffer controls and single stainings are shown. Bars indicate positive expression.
